# Intraoperative Three-Dimensional Transvaginal Ultrasound for Hysteroscopic Metroplasty: a Controlled Study

**DOI:** 10.1007/s43032-023-01277-x

**Published:** 2023-06-06

**Authors:** Ludovico Muzii, Giulia Galati, Giulia Mattei, Alessia Romito, Violante Di Donato, Innocenza Palaia, Giorgio Bogani, Roberto Angioli

**Affiliations:** 1 Department of Maternal and Child Health and Urological Sciences, Sapienza University of Rome, Rome, Italy; 2 Fondazione IRCCS Istituto Nazionale Dei Tumori Di Milano, Milan, Italy; 3 Department of Obstetrics and Gynaecology, Campus Bio-Medico, University of Rome, Rome, Italy

**Keywords:** Operative hysteroscopy, Recurrent pregnancy loss, Uterine septum, 3D ultrasound

## Abstract

This study aims to evaluate the role of intraoperative transvaginal three-dimensional ultrasound (3DUS) during hysteroscopic metroplasty. This is a prospective cohort of consecutive patients with septate uterus undergoing hysteroscopic metroplasty with intraoperative transvaginal 3DUS guidance compared to a historical control group of patients undergoing hysteroscopic metroplasty without 3DUS. We conducted our research in a tertiary care university hospital in Rome, Italy. This study involved nineteen patients undergoing 3DUS-guided hysteroscopic metroplasty for recurrent abortion or infertility compared to 19 age-matched controls undergoing metroplasty without 3DUS guidance. During hysteroscopic metroplasty, 3DUS was performed in the study group when the operator considered the procedure to be completed, according to standards of operative hysteroscopy. If 3DUS diagnosed a residual septum, the procedure was continued until a 3DUS diagnosis of a normal fundus was obtained. The patients were followed with a 3DUS performed 3 months after the procedure. The numbers of complete resections (residual septum absent), suboptimal resections (measurable residual septum of less than 10 mm), and incomplete resections (residual septum > 10 mm) in the intraoperative 3DUS group were compared to the numbers in the control group with no intraoperative 3DUS. At follow-up, measurable residual septa were obtained in 0% of the patients in the 3DUS-guided group versus 26% in the control group (*p* = 0.04). Residual septa of > 10 mm were obtained in 0% of the 3DUS group versus 10.5% in the control group (*p* = 0.48). Intraoperative 3DUS reduces the incidence of suboptimal septal resections at hysteroscopic metroplasty.

## Introduction

Congenital uterine malformations have an incidence rate of 8% in infertile women, among which the septate uterus is the most common [1]. The estimated prevalence is around 0.2–2.3% in women of reproductive age. A septate uterus may be associated with poor reproductive prognosis, such as infertility, recurrent pregnancy loss (RPL), preterm delivery or foetal malpresentation [2].

Diagnostic criteria, in particular for normal/arcuate/septate uterus, are one of the most debated topics in gynaecology since the classification parameters have often been changed over the years [3–7].

Hysteroscopic metroplasty has long been considered the gold standard treatment of uterine septum, in case of RPL or infertility, although evidence mostly derive from non-randomized, controlled and low-quality studies [2]. Given the lack of strong evidence, current guidelines do not give standardized recommendations: the ASRM guidelines suggest removing the septum [5], while other guidelines do not advise performing surgery unless within a clinical trial [8–10]. Moreover, the latest and larger publications have suggested reconsidering intervention as no differences in reproductive outcome were recently reported comparing expectant management and septum resection [11, 12].

One of the most problematic issues with hysteroscopic metroplasty is to decide when the resection should be stopped during the surgical procedure. If the procedure is stopped too early, a residual septum may be left and potentially impact on subsequent reproductive performance. On the other end, if the procedure is carried beyond the ideal limit, a perforation may ensue during surgery, or the uterus may rupture during subsequent pregnancies.

From a hysteroscopic point of view, the procedure of metroplasty is considered complete when the hysteroscope can be moved freely from one cornual recess to the other or when both tubal ostia can be viewed simultaneously [13–19]. Occurrence of bleeding from small vessels of the fundal myometrium [18], or the appearance of vertically running blood vessels during operative hysteroscopy [17, 19], have been considered additional criteria for terminating the procedure. However, even when the above criteria are followed, a residual septum may be evident at follow-up in 4% to 44% of the cases [16, 19]. If also minimal fundal notches are considered, i.e. between 1 and 10 mm, incomplete procedure rates as high as 61% have been reported [2].

Three-dimensional ultrasound (3DUS) has been demonstrated as a reliable diagnostic means to correctly diagnose uterine septa and to accurately differentiate between the septate and arcuate uterus [20, 21].

In the present study, we prospectively evaluated patients with a septate uterus, in which transvaginal 3DUS was utilized during operative hysteroscopy for septum resection to monitor the procedure in the operating room and confirm the completeness of the procedure. We aimed to determine whether concurrent 3DUS during surgery may improve the effectiveness of hysteroscopic metroplasty compared to a control group of age-matched patients undergoing the procedure without 3DUS monitoring, with attention to rates of residual septa post-surgery.

## Materials and Methods

All women consecutively referred to the Department of Obstetrics and Gynecology of Sapienza University and Campus University of Rome, Italy, complaining of RPL or infertility, and with a diagnosis of a complete or partial uterine septum, were included in the present study. Patients were excluded if, at preoperative diagnostic work-up, associated uterine pathological conditions were suspected (endometrial polyps, myomas, synechiae, endometrial hyperplasia, cervical or endometrial cancer).

**Fig. 1:**
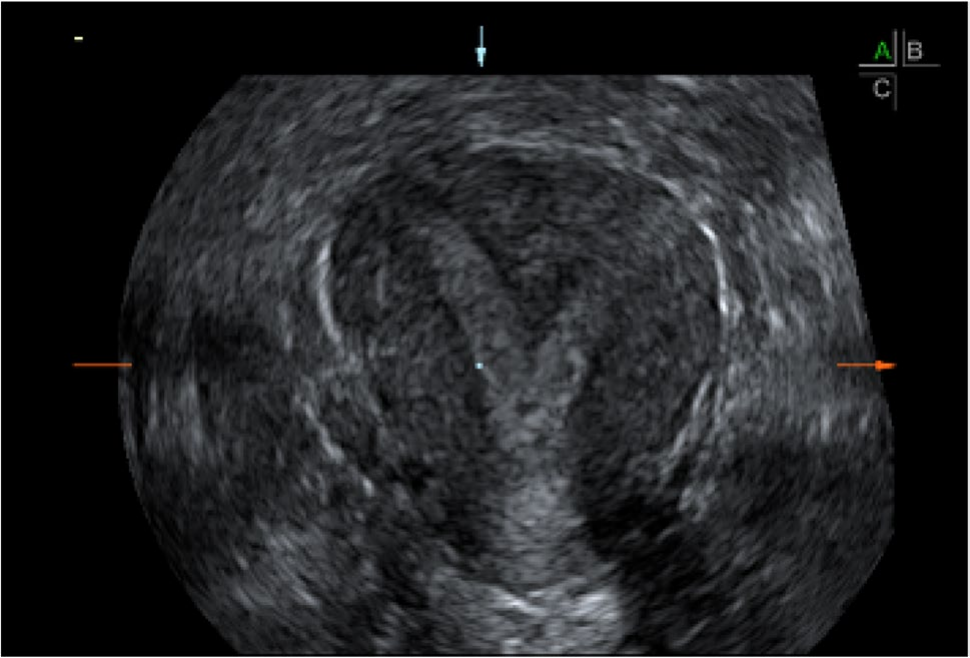
Septate uterus as seen on a coronal plane on 3DUS

The diagnosis of the uterine septum was confirmed with a transvaginal 3DUS scan performed in the secretory phase of the cycle (between day 17 and 25) immediately preceding the planned surgery (Fig. 1), with commercially available ultrasound equipment (GE Voluson E6, transvaginal 7 MHz volume probe, with 3D scan, GE Healthcare, Milwaukee, WI, USA). Women with amenorrhea or irregular cycle were examined regardless of the day of the cycle, outside the menstruation. A 3D volume of the uterus was obtained, in a sagittal view of the uterus, with a maximum sweep angle of 120° degrees and a 90° degrees angle between the ultrasound beam and the uterine axis. Images were stored and then rendered with volume contrast imaging (VCI).

A septate uterus was defined according to ASRM classification [5]. All procedures were carried out by a single operator (LM).

Diagnostic hysteroscopy was always performed immediately before surgery, with a vaginoscopic approach under general sedation. A 5-mm diameter continuous-flow hysteroscope with an oval profile, a 30° fore-oblique telescope and a 5 Fr operating channel (Office Continuous Flow Operative Hysteroscopy ‘size 5’; Karl Storz, Tuttlingen, Germany) was used with saline solution (NaCl 0.9%) as the distension medium. A bipolar electrode was used to remove the septum.

**Fig. 2:**
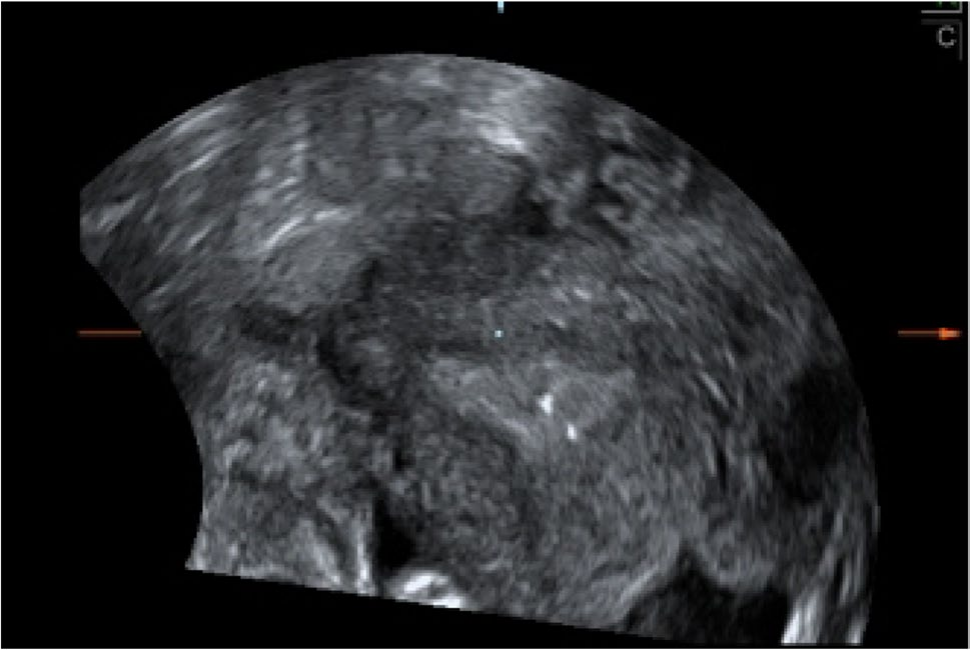
Intraoperative 3DUS demonstrates no residual septum, confirming the completeness of the procedure

Septum resection was carried out until the hysteroscope could be moved freely from one cornual recess to the other and when both tubal ostia could be viewed simultaneously. The procedure was temporarily stopped if bleeding from small vessels of the fundal myometrium occurred or when vertically running blood vessels were visible. When the surgeon considered the procedure to be completed according to the conventional hysteroscopic criteria outlined above, intraoperative transvaginal 3DUS was performed after removing the hysteroscope from the uterine cavity. If 3DUS diagnosed a residual septum defined as any measurable septum remnant, the procedure was continued until a 3DUS diagnosis of a normal fundus was obtained (Fig. 2).

The patients were followed with a 3DUS performed in the secretory phase (between day 17 and 25) 3 months after the procedure. Patients were compared to an age-matched group of patients who had undergone the same hysteroscopic procedure without 3DUS intraoperative monitoring. The numbers of complete resections (residual septum absent), suboptimal resections (measurable residual septum of less than 10 mm) and incomplete resections (residual septum > 10 mm) in the intraoperative 3DUS group were compared to the control group with no intraoperative 3DUS.

### Statistical Analysis

Continuous variables were summarized by mean and standard deviation (SD), and differences between variables were analysed performing the Mann–Whitney *U* test or the *t*-test, according to the normal distribution of data. The normality of data was assessed with Kolmogorov–Smirnov test. Categorical variables were represented by absolute frequencies and percentages (%). Comparisons between categorical variables were performed with the chi-square or Fisher’s exact test, as appropriate. The level of significance was set at α = 0.05. All analyses were performed using SPSS version 25.0.

## Results

Nineteen patients with a complete or partial uterine septum complaining of RPL or infertility were included in the present study. The study group undergoing hysteroscopic metroplasty with intraoperative 3DUS was compared to a control group of age-matched patients who had undergone the same procedure without intraoperative 3DUS in the preceding year. The mean patient age was 34.2 ± 4.5 years (mean ± SD) in the study group versus 33.9 ± 4.9 years in the control group (*p* = not significant). Indication for surgery was RPL or infertility in all patients.

The time of the surgical procedure was 18.2 ± 7.1 min in the study group versus 17.7 ± 8.3 min in the control group (*p* = not significant). In the study group, a mean additional time of 8.5 min was needed to complete the 3DUS scans.

The procedure was considered completed in all cases. No intraoperative or postoperative complications occurred in both groups.

**Fig. 3:**
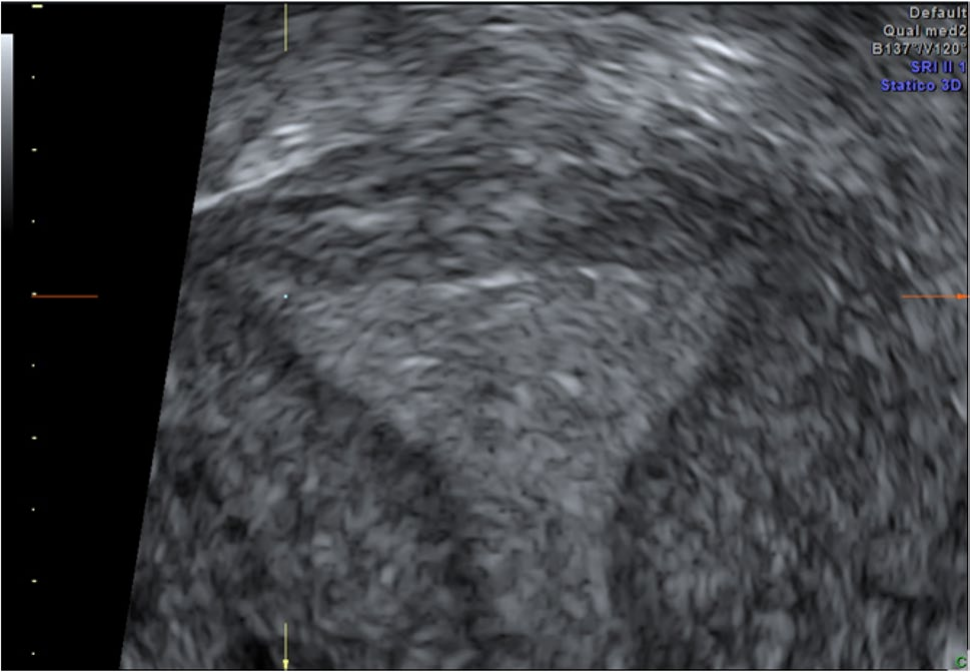
3DUS at 3-month follow-up confirms the absence of residual septum

At the 3DUS scan performed at follow-up 3 months after surgery (Fig. 3), measurable residual septa were diagnosed in 0% of the patients in the 3DUS-guided group versus 26% (5 of 19) in the control group (*p* = 0.04). Residual septa of > 10 mm was diagnosed in 0% of the 3DUS group versus 10.5% (2 of 19) in the control group (*p* = 0.48).

## Discussion

Hysteroscopic metroplasty is the gold standard for the treatment of septate uterus. Despite recent publications have shown no benefit of surgical treatment compared to expectant management [11, 12], the scientific community is still strongly convinced of its usefulness based on previous observational studies [22], although the urgent need of well-designed trial is unequivocal [23].

Hysteroscopic surgery aims to achieve a wide cavity since one of the reasons for miscarriage or preterm live birth was traced back to the altered uterus morphology or to the embryo implantation at the septum [24]. Whichever surgical technique is used, the most crucial issue of hysteroscopic metroplasty is to decide when to stop the procedure. The procedure may be incomplete, if the surgeons stop for the fear of perforation before the removal of the septum is complete, or, on the other hand, the procedure may be complicated by uterine perforation if the procedure is brought beyond the usual limit. There is no agreement between different operators as to which technique should be used to monitor the septum resection procedure. In most cases, traditional criteria, such as free movement from one cornual recess to the contralateral one, simultaneous view of both tubal ostia or bleeding vessels from the fundus, especially with lower intrauterine pressures, are followed. These criteria are, however, subjective, adding further uncertainty as to when to consider the procedure complete. Even when following the traditional criteria to set the limit for terminating the procedure, a residual septum may be evident at postoperative follow-up in 4% to 44% of the cases [16–19] or even more commonly when considering a notch between 1 and 10 mm [2]. On the other hand, uterine perforations, possibly due to a surgical procedure that has been brought beyond what is necessary, have been reported in 1.5–1.8% of the cases [2].

Some authors have reported the possibility of monitoring the hysteroscopic metroplasty during surgery, in the attempt to reduce the occurrence of incomplete procedures, without jeopardizing the results with higher perforation rates thus facilitating the identification of when to stop. Intraoperative monitoring has been reported with transabdominal US [25, 26] and transrectal US [27, 28] or with a graduated intrauterine palpator [29]. No report has been published so far on the possibility of using transvaginal 3DUS intraoperatively to monitor the procedure. The possible advantages of the transrectal route with respect to the transvaginal route is the need, with the latter procedure, to retrieve, and reinsert afterwards, the operative hysteroscope during the US procedure. With the transrectal procedure, the hysteroscope may be left inside the uterine cavity while performing the US evaluation, thus possibly shortening the operative time. However, performing the transrectal US procedure with the hysteroscope inside the uterus may on the other hand be more cumbersome, since both the surgeon and the sonographer must be on the same side of a limited working field. The transabdominal route may obviate the need to retrieve the hysteroscope and allows the two operators to work more comfortably in two different locations with respect to the patient. However, US performed through the transabdominal route details with lower resolution the pelvic structures, compared to the transvaginal or transrectal route, and is therefore not the standard procedure to evaluate the uterine anatomy.

According to a study by Coccia et al., intraoperative transabdominal US guidance may permit a more extensive resection beyond the limit conventionally defined by hysteroscopy, ensuring the same safety and efficacy of laparoscopic guidance. With the use of transabdominal US during the procedure, the authors reported no need for repeated procedures [25]. Accordingly, a study by Vigoureux et al. demonstrated that a persistent septum of > 10 mm was present after hysteroscopic metroplasty in 18% of the cases with intraoperative transabdominal US monitoring versus 39% of the cases when US guidance was not used during surgery (*p* = 0.04) [26].

Two authors reported on the use of transrectal 3DUS to monitor the metroplasty procedure [27, 28]. Kamel et al. reported a randomized clinical trial (RCT) where a 13% rate of residual (> 5 mm) septa were reported in 30 patients randomized to the conventional technique versus 0% in 30 patients randomized to intraoperative US monitoring (*p* = 0.04). Accordingly, Ghirardi et al. reported 4% of suboptimal (5–10 mm) or incomplete (> 10 mm) resections in 27 patients with intraoperative transrectal US monitoring versus 28% in 18 patients without monitoring (*p* = 0.03) [28].

Monitoring of the metroplasty during surgery may be performed also without any intraoperative imaging technique. Di Spiezio et al. demonstrated that the use of a novel graduated intrauterine palpator to measure the portion of the removed septum during the procedure, combined with presurgical evaluation with 3D-TVS, could provide a complete removal of a uterine septum, without requiring a second surgical attempt. The authors reported in a RCT a higher rate of complete procedures (71.5%) when using the intrauterine palpator during surgery versus the traditional procedure (41% of complete procedures, *p* = 0.006) [29]. Therefore, intraoperative monitoring with US, be it either with a transrectal or transabdominal probe or with intraoperative measurements with a uterine sound, has consistently been proven to reduce residual septa after surgery [25–29].

It is unclear if a minimal residual septum could have an impact on reproductive prognosis [18].

Since few observational studies have shown a 40% improvement of delivery rate after a second-step treatment for a residual septum, in clinical practice, patients are generally retreated if residual septum is seen [19]. Minimal residual septa have been traditionally considered not associated with worse reproductive prognosis. Fedele et al. reported on a series of 17 patients with a residual septum of 5 to 10 mm after operative hysteroscopy and compared the reproductive outcome of these patients with a group of 51 patients in which the procedure was considered complete, i.e. with no residual septum, or with a septum of less than 5 mm. Cumulative pregnancy rates at 18 months after the procedure were 44.5% in the group with a residual septum (5 to 10 mm) and 52.7% in the group with no residual, or < 5 mm, septum (*p* = not significant). Therefore, according to Fedele et al., a residual septum of less than 10 mm after hysteroscopic metroplasty has no significant impact on following reproductive outcome [18].

On the other hand, with results at variance with those of Fedele et al., Kormanyos et al. reported on a group of 94 patients with a septate uterus who underwent hysteroscopic resection. The septum was completely removed at first surgery in 58 patients (62%), whereas a residual septum was present in the remaining 36 patients (38%). In 35 of the 36 patients, the residual septum was less than 10 mm, whereas in one patient, the septum was more than 10 mm. Subsequent delivery rates after a minimum follow-up of 2 years were 44.8% in the group of 58 patients with a normal uterus after the first procedure and 19.4% in the 36 patients with a residual septum after the procedure (*p* < 0.05). Patients with a residual septum who did not conceive were submitted to a second procedure to treat the residual septum. After the second procedure aimed at normalizing the uterine cavity, 18 of these 29 patients (62.1%) achieved a pregnancy and delivered, a statistically significant difference compared to the 19.4% delivery rate for patients with a residual septum. Therefore, according to these data, even residual septa of less than 10 mm significantly impact on subsequent prognosis [19]. Although there is no consensus if a residual septum of less than 10 mm may or not impact on subsequent reproductive outcome, and in particular if a second surgery is needed to correct a residual septum after surgery, it comes without saying that the aim of obtaining a complete procedure with a single surgery without the need of a second intervention should be pursued. Data on reproductive outcomes in women with or without residual uterine septa are not available for the studies with intraoperative monitoring of the procedure [25–29]. Further studies are needed to better clarify whether small residual septa impact reproductive outcomes.

In the present study, the use of intraoperative transvaginal 3DUS significantly reduced the rate of incomplete procedures compared to a group of patients in which intraoperative 3DUS was not used. Recently, the use of 3DUS as support for hysteroscopy is growing, being it an easy, largely accessible and relatively low-cost diagnostic tool; 3DUS has been demonstrated to have high sensitivity and specificity in the detection of uterus malformation [30, 31].

A modern operating room (OR) may be equipped with intraoperative 3D ultrasound imaging. However, this requires additional costs, such as having trained staff and an ultrasound equipment. The latter can be used by multiple disciplines, which helps offset the cost. Indeed, with the advent of minimally invasive surgery, intraoperative imaging has become crucial to guaranteeing minimal invasiveness and maximal safety in different fields (urology, abdominal surgery, breast surgery etc.) [32]. Furthermore in gynaecology, using the ultrasound imaging in the OR may have several applications (endometriosis, uterine fibroids etc.) to optimize the chances of successful surgery. Coccia et al. reported a surgical excision of rectus abdominis muscle endometriosis with the advantage of intraoperative ultrasound to correctly identify the excision margins and entirely remove the lesion [33]. Moreover, several studies showed the benefit of intraoperative ultrasound applied during surgery for uterine fibroids. In particular, the US assessment improves residual myoma detection and enucleation following open or laparoscopic myomectomy [34, 35].

In our study, the rate of incomplete procedures with the use of intraoperative 3DUS was 0% compared to 26% in the control group (*p* < 0.05). When considering only residual septa of more than 10 mm, the rate of incomplete procedures was 0% in the study groups and 10.5% in the control group. The latter difference, although not statistically significant, may be clinically relevant and deserves confirmation in studies with larger sample sizes. This study also evaluated the time of the surgical procedure in both arms, reporting a mean additional time of 8.5 min in the study group. The few extra minutes required to perform the 3DUS scans can be deemed devoid of clinical impact on patients and consequences for operating room management. Finally, no intraoperative or postoperative complications were reported, demonstrating the safety of metroplasty when performed by an expert surgeon.

Some limitations of our study should be acknowledged. Firstly, our study is not randomized, and therefore, our results are inevitably exposed to possible confounders. RCTs are required to properly address this issue.

Secondly, our sample size was relatively small (only 38 women included) hampering robust conclusions. However, the comparison of the rate of incomplete procedures in the two groups reached statistical significance, allowing us to consider the intraoperative 3DUS septum evaluation as potentially useful.

Thirdly, the present study does not provide data on reproductive outcomes. Indeed, the short follow-up period — only 3 months — did not allow us to obtain data on pregnancy and live birth rates.

The results of this study should be interpreted by taking into account the limitations and underscoring the need for further studies with a longer follow-up period and a larger sample size.

## Conclusions

Our results support the potential usefulness of intraoperative transvaginal 3DUS in hysteroscopic metroplasty, reducing the rate of incomplete procedures, and potentially second-step surgeries. Larger studies are needed to confirm our results.

## Data Availability

All data involved in this study will be made available by the corresponding author upon request.
